# Quality Improvement Efforts in VA Community Living Centers Increased Following Public Reporting of Performance

**DOI:** 10.1093/geroni/igab046.077

**Published:** 2021-12-17

**Authors:** Heather Davila, Whitney Mills, Valerie Clark, Christine Hartmann, David Mohr, Dan Berlowitz, Amy Baughman, Camilla Pimentel

**Affiliations:** 1 Iowa City VA Healthcare System; University of Iowa Carver College of Medicine, Iowa City, Iowa, United States; 2 Providence VA Medical Center, Providence VAMC/Brown University, Rhode Island, United States; 3 VA Bedford Healthcare System, Bedford, Massachusetts, United States; 4 VA Bedford Healthcare System, VA Bedford Healthcare System, Massachusetts, United States; 5 VA Boston Healthcare System, Boston, Massachusetts, United States; 6 UMass-Lowell, UMass-Lowell, Massachusetts, United States; 7 Massachusetts General Hospital, Massachusetts General Hospital/Boston, Massachusetts, United States

## Abstract

In 2018, the US Department of Veterans Affairs (VA) began publicly reporting performance ratings for its 134 Community Living Centers (CLCs; nursing homes) based on health inspections, staffing, and clinical quality measures. CLCs operate within a large, integrated healthcare system with unique financial and market incentives. Although public reporting has led to quality improvements in non-VA nursing homes, we do not know whether CLCs respond to public reporting differently than private sector nursing homes. To address this knowledge gap, we used a comparative case study approach involving 3 purposively selected CLCs with varied (low, medium, high) performance ratings. We conducted semi-structured interviews with personnel (n=12) responsible for quality measurement and improvement. Interviews focused on opinions of public reporting, actions taken to improve performance ratings, and motivations for change. Participants indicated public reporting improved transparency and provided an “outside perspective” on their performance. Strategies to improve performance ratings involved 1) data/information, 2) individual roles, and 3) teamwork/communication. All 3 CLCs made changes in these areas, yet respondents in the higher performing CLCs described implementing more strategies immediately after learning their ratings. Respondents in all 3 CLCs described being motivated to deliver good care and achieve public ratings that reflected the care they provided. This meant addressing internal weaknesses that contributed to lower scores for 2 CLCs. Our findings suggest public reporting may improve internal data collection, reporting, and quality improvement efforts in CLCs. They highlight the potential positive impact of public reporting in prompting quality improvement in nursing homes.

